# Salivary 1,5-Anhydroglucitol and Vitamin Levels in Relation to Caries Risk in Children

**DOI:** 10.1155/2019/4503450

**Published:** 2019-02-07

**Authors:** Sadatullah Syed, Syed M. Yassin, Ali A. Dawasaz, Mohammed Amanullah, Ibrahim Alshahrani, Rafi Ahmad Togoo

**Affiliations:** ^1^Department of Diagnostic Sciences and Oral Biology, King Khalid University College of Dentistry, Abha, Saudi Arabia; ^2^Department of Pediatric Dentistry and Orthodontic Sciences, King Khalid University College of Dentistry, Abha, Saudi Arabia; ^3^Department of Biochemistry, King Khalid University College of Medicine, Abha, Saudi Arabia

## Abstract

The objective of this study was to evaluate the association between salivary 1,5-anhydroglucitol (AG), vitamins A (VA), C (VC), and E (VE), and caries risk in children. 100 healthy children aged between 6 and 13 years were divided into two equal groups of caries-free (DMFS/dmfs=0) and caries active (DMFS/dmfs>3). Unstimulated midmorning saliva was collected from all the children and the levels of salivary AG and vitamins A, C, and E were measured. Caries risk assessment was done using American Academy of Pediatric Dentistry Caries Assessment Tool. Analysis of salivary AG and vitamins was performed using a commercially available ELISA kit. Low levels of AG were present in caries active and high caries risk groups compared to caries-free and low/medium caries risk groups. This difference is statistically significant (p < 0.05). A strong negative correlation between AG and caries activity was observed in the caries active group. VA was not related to caries activity, while VC and VE displayed a statistically significant correlation (p < 0.05). Similarly, a strong negative correlation was observed between the levels of AG and high caries risk group. Salivary AG, VC, and VE together are related to caries risk in caries active children. These salivary parameters can act as indicator of caries status in children.

## 1. Introduction

Dental caries is defined as a common chronic, multifactorial, infectious disease that results in the demineralization and destruction of teeth [1]. It is the most prevalent chronic disease affecting 80% of the human population and 50% of schoolchildren and remains a major health issue in developed as well as developing countries [2]. An estimated 23% of American children's, over 60% of Chinese children's, and 80% of Saudi children's primary teeth are affected by dental caries [3–5]. The treatment of dental diseases leads to a huge economic impact costing billions of dollars globally [6]. WHO Oral Health Program report 2003 acknowledged dental caries as a major public health problem and emphasized on promoting its prevention before its manifestation [7]. However, methods that can accurately predict the manifestation of caries risk in individuals are still not available. Caries risk assessment tools are methods that address the probability of the occurrence of new cavitated/incipient lesions, or any change in the size and activity of already existing lesions [8]. Although the most reliable predictor of future caries status is based on caries history, it is not particularly useful in children [9]. The American Academy of Pediatric Dentistry Caries Risk Assessment Tool (AAPD CAT) is regarded as a sensitive and practical tool in assessing caries risk in children [10]. The AAPD CAT classifies caries risk as low, moderate, and high based on a child's age, biological factors, protective factors, and clinical findings.

Whole saliva is a complex biological fluid that plays a crucial role in maintaining the oral ecosystem. It contains antimicrobial and buffering mechanism that protects and maintains the teeth. The other important defense mechanism of saliva against dental caries is its antioxidant system. The system comprises of enzymes (peroxidase, catalase, superoxide dismutase, and glutathione peroxidase) and small molecules (uric acid, vitamins E and C) [11, 12]. The total antioxidant capacity (TAC) of saliva is higher in caries active children and increases with increased caries activity, and this has been considered by some as a caries predictor [13].

Dietary sugars, dental plaque bacteria, and teeth form the triad of etiology for dental caries. Dietary sugars, such as sucrose, are major contributing factors to the etiology of caries. Cariogenic bacteria metabolize sucrose producing acid by-products leading to the demineralization of dental enamel. Additionally, sucrose facilitates the production of extracellular polysaccharides that helps in the adherence of plaque and the plaque microorganism to tooth surface [14]. On the other hand, noncariogenic sugar alcohols such as sorbitol, xylitol, mannitol, and erythritol do not cause a decrease in pH in dental plaque and they are believed to induce growth inhibition of cariogenic bacteria [15, 16].

Like the aforementioned polyols, 1,5-anhydroglucitol (AG) is a metabolically inert monosaccharide present in the organs and tissues of the human body. Chemically, it is known as 1,5-anhydro-D-glucitol, a major circulating cyclic polyol, with a molecular formula of C_6_H_12_O_5_, and an average molecular mass of 164.156 Daltons. It is a noninvasive marker for glycemic control and is inversely associated with blood glucose concentration. The levels of salivary AG are positively correlated to levels of AG in blood; hence, salivary AG is an equally effective marker for short-term glycemia [17]. Metabolomic profiling of carious children has revealed a significant decrease in salivary saccharides using nuclear magnetic resonance imaging technique [18].

Levels of salivary AG have not been investigated in relation to caries activity and various levels of caries risk in children. Therefore, the research questions in this study were whether the levels of salivary AG and vitamins A (VA), C (VC), and E (VE) are related to caries activity and caries risk in children. With this perspective, the objective of the study was to evaluate the association of salivary AG, VA, VC, and VE in caries active and caries risk children.

## 2. Materials and Methods

### 2.1. Study Design and Sampling

This cross-sectional study was conducted on healthy one hundred male and female children of age group between 6 and 13 years reporting to pediatric dental clinics, King Khalid University College of Dentistry. The sample size was determined according to the statistical formula used in previous similar studies [19].

(1) $$\begin{array}{c} n={\left(\;1.96\;\right)}^{2}\times p\times \frac{\left(1\text{-}p\right)}{d^{2}} \end{array}$$

where n is sample size, p is prevalence = 50%, and d is margin of error = 15

Therefore, the minimum sample size required for test and control group was 41.83. Following ethical approval from Institutional Review Board (SRC/ETH/2017-18/051), parents of subjects providing informed consent were included in the study. Children on medications affecting salivary secretions, medically compromised children and those with arrested carious lesion were excluded. The selected children were divided into two groups, namely, caries-free and caries active groups. Children with DMFS/dmfs score more than 3 were considered caries active, while children with DMFS/dmfs score zero were considered caries-free.

An examination form was used to record the demographic details, medical history, diet history, oral hygiene practices, and DMFS/dmfs details. Dental caries was recorded as per World health organization 1997 criteria [20].

In order to investigate the correlation between salivary AG, VA, VC, VE, and caries risk, the subjects were also categorized into low, medium, and high caries risk groups as per AAPD CAT criteria [21]. The AAPD CAT considers the biological, protective factors as well as clinical findings for assessing caries risk. The data for biological and protective factors was obtained from the mothers and was recorded in the caries risk assessment form along with the clinical findings.

All the children were seen in the morning and saliva was collected 2 hours after breakfast using the passive drooling method [22]. The samples were coded by the principal investigator and transported to the laboratory in an icebox, where they were stored at -80°C. In the laboratory, the level of salivary AG, VA, VC, and VE were measured for all the samples.

### 2.2. Laboratory Procedures [23–25]

After all samples were collected, the stored saliva was thawed and centrifuged (Kenley, London, UK, rotor radius 7 cm) at 3500 RPM for 10 min to remove proteins; the clear supernatant was collected to estimate AG and VA, VC, and VE levels. Enzyme Linked Immunosorbent Assay (ELISA) kit for AG (product CEB046Ge, Cloud-Clone Corp Katy, TX, USA), Retinol/VA (product CED051Ge, Cloud-Clone Corp Katy, TX, USA), Ascorbic acid/VC (product CEA913Ge, Cloud-Clone Corp Katy, TX, USA), and Alpha Tocopherol/VE (product CEA922Ge, Cloud-Clone Corp Katy, TX, USA) was used with minor modifications of the protocol provided by the manufacturer. Because saliva contains minute quantities of these constituents, the standard solutions were diluted to very low concentrations; consequently, the quantity of saliva and reagents used were doubled. Briefly, the concentration of standard AG in the stock solution provided with the kit was 150 *μ*g/mL. This was diluted serially using the standard diluent solution (SDS) to obtain standard solutions with concentrations of 1.9 *μ*g/mL, 0.95 *μ*g/mL, 0.475 *μ*g/mL, 0.237 *μ*g/mL, 0.118 *μ*g/mL, 0.059 *μ*g/mL, 0.029 *μ*g/mL, and 0.00 *μ*g/mL. The concentration of the standard VA in the stock solution provided in the kit was 10,000 ng/mL. This was diluted with the SDS to obtain working standard solutions ranging from 370.4 ng/mL to 2.89 ng/mL (serial two-fold dilutions). The concentration of the standard VC in the stock solution provided with the kit was 40,000 ng/mL (4 mg/dL). This was diluted with SDS according to manufacturer's instructions. The concentration of the standard VE in the stock solution provided with the kit was 100 *µ*g/mL. This was diluted with SDS to obtain working standard solutions ranging from 33.33 ng/mL to 0.26 *µ*g/mL (serial twofold dilutions).

All other reagents from each of the kits were prepared separately. One hundred microliters of standard solution and saliva sample solution were pipetted into each of the wells of the ELISA plate, and 100 *μ*L of “prepared detection reagent A” from the kit was added immediately. This was mixed and incubated for 1 h at 37°C and later aspirated and washed three times. Later, 200 *μ*L of “prepared detection reagent B” was added and incubated for 30°min at 37°C; subsequently, it was aspirated and washed 5 times. To this, 180 *μ*L of substrate solution was added and incubated for 10-20 min at 37°C. Finally, 100 *μ*L of stop solution was added, and the optical density (OD) was read in an ELISA reader at 450 nm immediately. To make the calculation easier, the OD value of the standard (X-axis) was plotted against the log of the concentration of the standard (Y-axis). The best-fit straight line was drawn through the standard points, as determined by regression analysis, using plotting software curve expert version 2.6.4. The concentration of the biochemical constituents in the saliva samples was determined from each of these curves using the same software.

The resultant values of salivary AG and VA, VC, and VE, were statistically analyzed using Pearson's correlation, Student's* t*-test, analysis of variance (ANOVA), linear regression analysis, and Durbin Watson tests were performed using SPSS version 24.0 (IBM Corporation, Armonk, NY, USA) for Windows (Microsoft Corporation, Redmond, WA, USA). The p value of 0.05 or less was considered significant.

## 3. Results

Of the 100 saliva samples, laboratory procedures were completed in 92: five samples were discarded because of contamination, and three were excluded due to insufficient volume. There were 41 samples in caries-free group and 51 samples in caries active group. Likewise, the low, medium, and high caries risk groups had 25, 16, and 51 samples, respectively.

Table 1 shows the mean values of salivary parameters in all the caries groups. These values for AG, VA, VC, and VE were low in caries active group and high caries risk group.  Table 2 shows the correlation of salivary parameters in caries active groups and caries risk groups. As evident from the table, all the parameters have a negative correlation except VA. AG has the highest Pearson's correlation value of -.78 and -.91 in caries active groups and caries risk groups, respectively. This correlation is statistically significant (p < 0.05) for all the salivary parameters except VA.

Comparative analysis of means of the salivary parameters between the caries activity groups using t-test is presented in Table 3. There is a difference in the mean values of AG, VA, VC, and VE in caries-free and caries active groups, but this difference is statistically significant (p < 0.05) for AG, VC, and VE only.

Analysis of variance testing was performed to compare the mean levels of salivary parameters in low/medium/high caries risk groups. The analysis revealed a statistically significant (p<0.05) difference for salivary AG (Table 4). Unlike AG, none of the investigated salivary vitamins demonstrated a statistically significant difference with ANOVA testing (Table 3).

Linear regression analysis was performed to identify caries risk indicators among the salivary constituents. The base formula for linear regression analysis was Y=b0+b1x1+ b2x2+ b3x3+ b4x4+…+ bnxn. Caries risk groups (Y) were considered to be the dependent variables and salivary AG, VA, VC, and VE were the independent variables (X1, X2, X3, and X4, respectively). Following linear regression analysis, the correlation of caries risk with independent variables was 92.3%, indicating a strong positive correlation. The fitness of the above model was 85% based on R-square value (Table 5). In this time series, a Durbin Watson score of 1.41 suggests a positive correlation within the sample population.

## 4. Discussion

The grouping of study population was done on the basis of DMFS/dmfs and AAPD CAT. The advantage of using these tools is that it incorporates all accessible evidence including cavitated lesions, and protective and causative factors in assessing the levels of caries activity and caries risk. Grouping children as caries-free, caries active, and low, moderate, high caries risk on the basis of age, biological factors, protective factors, and nutritional and clinical findings enables a more comprehensive and evidence-based approach to caries assessment. Other tools, such as The International Caries Detection and Assessment System, have too many subgroups/codes, while the Nyvad criteria are more specific to the low caries group [26, 27].

The mean value of salivary AG in our study population was comparable with earlier reported salivary AG levels for healthy nondiabetic individuals [28]. It is accepted that covariates, such as age, sex, and ethnicity, have no significant effect on the levels of salivary AG; accordingly, our results appear to be consistent with the previous study. Khademi et al. [29] studied the salivary levels of VC and VE in healthy and Recurrent Aphthous Stomatitis patients. The normal values of VC (1.06 *μ*g/ml) and VE (11.29 *μ*g/ml) in their study were similar to our study (1.21 *μ*g/ml and 12.18 *μ*g/ml, Table 1). The mean value of VA in our study was lower than Khademi et al. [29] results.

Our results reveal the contribution of AG, VA, VC, and VE in caries activity and caries risk. An increase in AG value by 100% reduced the degree of caries risk by 113%; however, an increase in salivary vitamin A by 100% reduced the degree of caries risk by only 0.1%. This indicates the strong negative correlation between AG and caries risk groups. A moderate negative correlation between caries risk groups and VC was also recorded. In addition, a strong correlation between caries risk groups and salivary VE was observed. Collectively, only AG, VC, and VE are related to caries risk groups; therefore, AG draws maximum influence on caries risk, whereas VA has minimum influence. Considering all of the independent variables assessed in this study as possible factors, their influence on caries risk is as high as 84%, while the unknown factors not considered in this study may constitute the remainder. According to the results of Durbin Watson score, any bias arising due to the time of saliva collection is statistically ruled out.

Fidalgo T et al. [18] reported decreased salivary saccharide concentration in caries subjects. AG is a monosaccharide, which is completely nonmetabolized, and remains relatively constant in the blood and saliva. It is absorbed into the bloodstream and filtered in the glomeruli of the kidneys. It is reabsorbed back into the blood by the renal proximal tubules [30]. A small quantity equal to the amount ingested is released in the urine, thus maintaining its level in the body [31].

The exact pathophysiology of reduction of salivary AG in presence of caries activity is not clear; this can be the scope for further investigation. However, the results from our study and previous similar studies permits the hypothesis that an increased volume of cariogenic bacteria in caries active host utilizes the saccharide substrate for their cellular metabolism leading to reduced concentration of salivary saccharides [18]. It is not certain whether the cariogenic bacteria thrive on AG as they do on other saccharides present in saliva. Another assumption is that the salivary AG concentration is reduced in presence of increased level of fermentable carbohydrates, which are a strong causative factor of caries. This can be a possibility since Juraschek et al. [32] reported decreased plasma concentration of AG with increase dietary carbohydrates in healthy nondiabetic individuals. It can also be a combination of increased dietary carbohydrates leading to decrease in salivary AG and the cariogenic bacteria using the saccharides including AG for their energy metabolism further decreasing the AG concentration in saliva.

Presently, caries predictor tools are not able to accurately predict prospective caries activity in children [33]. However, caries history is the strongest factor influencing accuracy of caries risk prediction in multivariable regression models. Results of our study justifies the need of further investigations on salivary AG, VC, and VE as effective caries activity indicators.

## 5. Conclusion

We identified a strong negative correlation between the levels of salivary AG and high caries activity in children. The values of AG, in addition to VC and VE, were related to caries risk. These salivary parameters can be used as high caries activity and caries risk indicators in healthy children, although further investigations with longitudinal designs and larger sample sizes are warranted.

## Figures and Tables

**Table 1:** Mean values of salivary parameters.

		AG (*μ*g/ml)	VA (ng/ml)	VC (*μ*g/ml)	VE (*μ*g/100 ml)
Caries Free (N = 41)	Mean	1.43	80.46	1.53	13.28
	(±)	(0.61)	(63.01)	(0.81)	(2.28)

Caries Active (N = 51)	Mean	0.35	68.38	0.95	11.30
	(±)	(0.19)	(65.21)	(0.55)	(3.40)

Low Caries Risk (N = 25)	Mean	1.85	95.91	1.85	12.40
	(±)	(0.36)	(73.63)	(0.87)	(2.21)

Medium Caries Risk (N = 16)	Mean	0.76	56.33	1.04	14.66
	(±)	(0.09)	(29.89)	(0.36)	(1.68)

High Caries Risk (N = 51)	Mean	0.35	68.38	0.95	11.30
	(±)	(0.19)	(65.21)	(0.55)	(3.40)

Total sample (N = 92)	Mean	0.83	73.76	1.21	12.18
	(±)	(0.68)	(64.17)	(0.74)	(3.1)

**Table 2:** Correlation between salivary constituents of caries-free, caries active, and low, medium, and high caries risk groups using Pearson's correlation test.

		AG	VA	VC	VE
CF,CA groups^*∗*^	Significance	0.00	0.37	0.00	0.00
(N = 92)	Pearson's correlation coefficient	-.78	-.09	-.39	-.32

L,M,H groups^*∗*^	Significance	0.00	0.12	0.00	0.04
(N = 92)	Pearson's correlation coefficient	-.91	-.16	-.50	-.20

^*∗*^CF: caries-free, CA: caries active, L: low risk, M: medium risk, and H: high risk.

**Table 3:** Comparison of means of salivary constituents in caries-free and caries active groups using t-test.

	F	t	p-value
AG	148.41	11.82	0.00
VA	1.05	.89	0.37
VC	12.49	4.07	0.00
VE	2.93	3.20	0.00

**Table 4:** Comparison of means of salivary constituents in low, medium, and high caries risk groups using ANOVA.

		F	Sig.
AG	Between Groups	3.048	.033
	Within Groups		
	Total		

VA	Between Groups	1.078	.363
	Within Groups		
	Total		

VC	Between Groups	.847	.472
	Within Groups		
	Total		

VE	Between Groups	1.670	.179
	Within Groups		
	Total		

**Table 5:** Linear regression model using dependent variable (caries risk groups) and independent variables (salivary constituents).

Model Summary^b^										

Model	R	R Square	Adjusted R Square	Std. Error of the Estimate	Change Statistics					Durbin-Watson
					R Square Change	F Change	df1	df2	Sig. F Change	

1	.923^a^	.851	.845	.342	.851	124.681	4	87	.000	1.410

a. Predictors: (Constant), VE, AG, VA, VC										

b. Dependent Variable: Caries risk										

Coefficients^a^										

Model			Unstandardized Coefficients			Standardized Coefficients		t		Sig.
			B	Std. Error		Beta				

1	Constant		3.411	.162				21.022		.000
	AG		-1.133	.062		-.898		-18.374		.000
	VA		.001	.001		.040		.906		.367
	VC		-.053	.057		-.046		-.939		.350
	VE		-.013	.012		-.047		-1.061		.292

a. Dependent Variable: Caries risk										

## Data Availability

The data used to support the findings of this study are included within the article.
